# S81. Proffered paper: A new PD1-CD28 chimeric receptor overcomes PD-1-mediated immunosuppression in adoptive T cell therapy

**DOI:** 10.1186/2051-1426-2-S2-I19

**Published:** 2014-03-12

**Authors:** S Grassmann, P Peters, Y Zeng, J Schmollinger, S Endres, S Kobold

**Affiliations:** 1 Division of Clinical Pharmacology, Munich, Germany

## Background

Although tumour-specific cytotoxic T cells are capable of killing tumour cells both *in vitro* and *in vivo*, treatment with adoptive T cell transfer does not lead to sufficient tumour regression without adjuvant therapy. Tumour-promoted T cell exhaustion and anergy have been proposed to contribute to this lack of efficacy. We and others have previously shown that programmed death receptor-1 (PD-1) upregulation is a hallmark of tumour infiltrating, adoptively transferred T cells. PD-1 and its ligand (PD-L1) constitute a major immunosuppressive axis driven by tumour cells. Disruption of this axis may hit an Achilles heel of tumour immune escape.

## Material and methods

A PD1-CD28 chimeric receptor was cloned into the retroviral vector pMP71 and expressed in primary murine T cells specific for the model antigen ovalbumin (OT-1 cells). Functionality was addressed *in vitro* using ELISA and flow cytometry. *In vivo*, ovalbumin and PD-L1 overexpressing Panc02 cells (syngeneic pancreatic cancer cell line) were inoculated subcutaneously in immunocompetent female C57Bl/6 mice. Mice (n = 6 per group) were treated twice i.v. with PD1-CD28 chimeric receptor-transduced T cells or control T cells.

## Results

*In vitro*, PD-1-CD28 chimeric receptor-transduced primary T cells released 130 fold more interleukin-2 (IL-2) and 300 fold more interferon-γ than untransduced or control-transduced T cells when stimulated with CD3 and PD-L1, demonstrating the functionality of the chimeric receptor (p = 0.0014). In co-culture experiments with the Panc02 tumour cells, effective co-stimulation through PD1-CD28 was only seen in the presence of the TCR-recognized antigen ovalbumine and PD-L1. Upon blockade of MHC or PD-1, co-stimulation through the receptor was abrogated. Culture of transduced T cells in the presence of CD3 and PD-L1 increased cell numbers 4 fold and significantly increased viability of cells compared to untransduced or control-transduced T cells (p < 0.0001). *In vivo*, treatment of mice with an established (OVA and PD-L1 expressing) Panc02 subcutaneous tumour (mean tumour size at treatment onset 26 mm^2^) with PD1-CD28-transduced OT-1 slowed tumour growth compared to treatment with control-transduced OT-1 cells (p < 0.001). This demonstrates the functionality of the chimeric receptor in an immunocompetent organism.

## Conclusions

Adoptive T cells therapy with PD-1-CD28 chimeric receptor-transduced T cells is a promising approach to overcome PD-1-PD-L1-mediated tumour-induced anergy and immunosuppression.

